# Optical Gratings Coated with Thin Si_3_N_4_ Layer for Efficient Immunosensing by Optical Waveguide Lightmode Spectroscopy

**DOI:** 10.3390/bios2020114

**Published:** 2012-04-10

**Authors:** Lorena Diéguez, David Caballero, Josep Calderer, Mauricio Moreno, Elena Martínez, Josep Samitier

**Affiliations:** 1 Department of Electronics, University of Barcelona, C/Martí i Franquès 1, Barcelona, ES 08028, Spain; E-Mails: caballero@unistra.fr (D.C.); mmoreno@el.ub.es (M.M.); jsamitier@ibecbarcelona.eu (J.S.); 2 Nanobioengineering Group, Institute for Bioengineering of Catalonia (IBEC), C/Baldiri Reixac 10-12, Barcelona, ES 08028, Spain; E-Mail: emartinez@ibecbarcelona.eu; 3 Networking Research Center on Bioengineering, Biomaterials and Nanomedicine (CIBER-BBN), C/María de Luna 11, Edificio CEEI, Zaragoza, ES 50018, Spain; 4 Electronic Engineering Department, Universitat Politècnica de Catalunya, Campus Nord, Barcelona, ES 08034, Spain; E-Mail: calderer@eel.upc.edu

**Keywords:** silicon nitride, optical gratings, waveguide, biosensor

## Abstract

New silicon nitride coated optical gratings were tested by means of Optical Waveguide Lightmode Spectroscopy (OWLS). A thin layer of 10 nm of transparent silicon nitride was deposited on commercial optical gratings by means of sputtering. The quality of the layer was tested by x-ray photoelectron spectroscopy and atomic force microscopy. As a proof of concept, the sensors were successfully tested with OWLS by monitoring the concentration dependence on the detection of an antibody-protein pair. The potential of the Si_3_N_4_ as functional layer in a real-time biosensor opens new ways for the integration of optical waveguides with microelectronics.

## 1. Introduction

Biosensors are nowadays a powerful tool to enable the detection of biological interactions, as more sophisticated rapid measurements devices are necessary to collect real-time or in-line information from a variety of environments, from bioprocessing to healthcare [1]. In particular, the detection of low concentrated specific biomolecular targets in body fluids still remains a challenge in developing early prognosis and specific treatments. An analogous challenge is involved in the detection of biomolecules in environmental or biological weapons. 

Immunosensors are biosensors that use antibody-antigen interactions to provide high specificity, achieved by the molecular recognition of target analytes (usually the antigens) by antibodies to form a stable complex on the surface of the system [2,3]. A wide range of transducers have been explored for immunosensing such as electrochemical, piezoelectric or optical, with differences in sensor sensitivity and reproducibility [4]. In fact, it is recognized that to combine direct immunosensing with optical analysis is a great approach to achieve the best sensitivity and selectivity [5]. In this context, evanescent field optical biosensors constitute a label free sensing instrument that measures the variation of the refractive index of the adsorbed layer onto a chip surface and translate this variation into surface concentration of the adsorbed molecule [6]. In the field of optical label-free biosensing, the most used transducer is a sensor chip with gold surface (Surface Plasmon Resonance), which presents a relatively easy functionalization and provides information about the success of the biomolecular adsorption on the surface and the recognition events [7]. Depending on the application, the possibility of choosing the material of the active sensing surface would remain a challenge. Recently, as an alternative to Surface Plasmon Resonance technique, the Optical Waveguide Spectroscopy technique, a grating coupler optical biosensor, has emerged [8]. In this system it is possible to simulate both the refractive index change and the thickness of the adsorbed film and its mass by numerical methods, exhibiting a very high sensitivity of 1 ng/cm^2^ [9]. The system keeps this sensitivity measuring changes at the sensor surface closer than 200 nm [10]. In opposition to SPR, in the case of the grating couplers the sensing substrate is not a metal, but a transparent material to allow the light coupling in the waveguide. Dielectrics and conductors are normally used as elements of the sensing waveguide, but also the grating-coupler waveguide sensors can be covered using thin layers of SiO_2_, Ta_2_O_5_ and SiO_2_/TiO_2_ without affecting their sensitivity and allowing simulation of the surface properties of the material of interest [11,12]. Even electrically conductive, transparent oxide layers, such as ITO, can be applied as coatings on the grating coupler sensor chip. With such materials, the grating-coupler sensor can be combined with an electrochemical sensor, opening up new fields of applications [13]. 

Table 1 shows a comparison of the most common insulators. As shown in the table, although the oxides are all widely used as dielectrics in Field Effect Transistors, nitride films provide a good compromise with low leakage current and low conductivity [14]. Also, silicon nitride has been broadly exploited in optical waveguides and preferred than silicon oxide, due to its high refractive index [15,16]). It also possesses a number of fabrication advantages such as the absence of undesirable impurities and the good control of the film composition and thickness. This is especially important for ultrathin layers used in optical spectroscopy measurements. Regardless of the performance of silicon nitride as an insulator, the aim of this work is to provide a technology that will allow quantifying the adsorption of biomolecules onto the gate of nitride-based transistors.

**Table 1:** Properties of thin layers of various amorphous insulators.

	Si_3_N_4_	SiO_2_	TiO_2_	Ta_2_O_5_	HfO_2_
Refractive index	2.02 [16]	1.46 [16]	2.58 [17]	2.10 [12]	2.08 [18]
Leakage current (A/cm^2^) at 2 V	1 × 10^−15^ [19]	1 × 10^−8^ [19]	>1 × 10^−7^ [20]	9 × 10^−8^ [21]	2 × 10^−9^ [18]
Dielectric constant	7.5 [14]	3.9 [14]	80–30 [20]	26.0 [21]	25.0 [18]

Although silicon nitride-based immunosensors are widely reported [22,23,24], this is the first time that commercial optical gratings are coated with a thin layer of silicon-nitride to allow real-time quantitative studies of the absorption of biomolecules onto its surface, to be used in calibration of other techniques, such as Field Effect Transistor based biosensor. In this way, the non-specific adsorption of an analyte onto the insulator layer could be estimated and later subtracted. This work presents advances in the fabrication of new biomaterials for optical biosensors by demonstrating the potential of the Si_3_N_4_ as functional layer in a real-time biosensor, and opening new ways for the integration of optical waveguides with microelectronics.

## 2. Experimental Section

### 2.1. Materials and Reagents

Silicon-titanium oxide grating substrates (depth 20 nm, period 2,400 lines/mm) were supplied by MicroVacuum Ltd (Budapest, Hungary) and consist on a 170 nm thick SiO_2_-TiO_2_ oxide layer (n_f_ = 1.77) onto a glass substrate (n_s_ = 1.53). All refractive indices have been measured at 632.8 nm. The grating area dimensions were 2 mm in width per 12 mm in length on a total chip size of 12 mm in length, 8 mm in width and 0.5 mm thick. Previously to be coated with silicon nitride, the gratings were thoroughly cleaned with organic solvents and Milli-Q water. 

Sodium cyanoborohydride, Sodium hydroxide, Ethanolamine, Glycine and HSA (Albumin solution from human serum 30% in 0.85% sodium chloride) were purchased from Sigma-Aldrich (USA). The triethoxysilane aldehyde (TEA), which was obtained from United Chemical Technologies (USA), was stored in the dark, under argon and at room temperature. Anti-HSA rabbit monoclonal antibodies (IgG) having a molecular weight of about 150 kDa were purchased from AntibodyBcn (Spain). Antibodies were prepared in phosphate buffer saline solution (0.01 M phosphate buffer, 0.0027 M potassium chloride and 0.137 M sodium chloride, PH 7.4). Hydrochloric acid was purchased from Merck (Germany). 

### 2.2. OWLS Transducer and Measuring Principle

**Figure 1:**
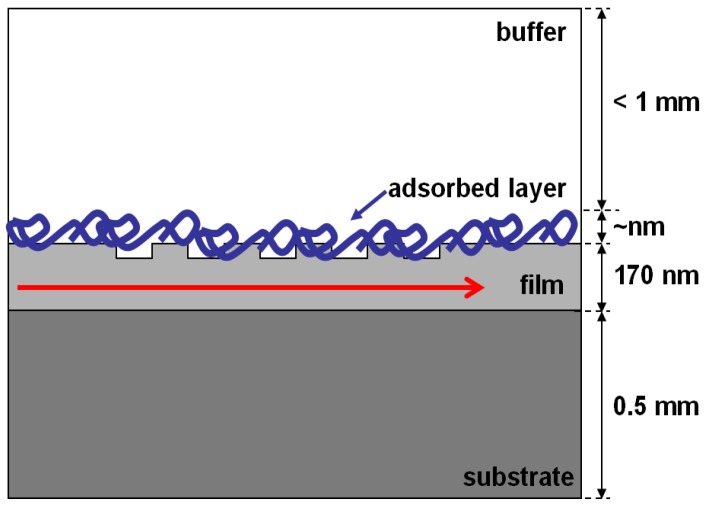
Grating coupler scheme. The molecules attached to the surface form the adsorbed layer. This new layer changes the in-coupling conditions, allowing the detection.

The structure and measuring principle of the optical grating coupler sensor chip are depicted in Figure 1. A He–Ne laser (λ = 632.8 nm) is diffracted by the grating and, at a characteristic incident angle, there is a constructive interference (phase shift of the internal reflection is zero) that excites a guided mode, then the light propagates through the waveguide via total internal reflection and an evanescent field is generated onto the covering medium. 

The change in the refraction index of the covering layer produced by the analyte recognition can be monitored on line by measuring the changes in the incoupling angle [10,25,26]. In this case, a commercial OWLS instrument from MicroVacuum Ltd. (Budapest, Hungary) was used. This system software measures the effective refractive index on the zero transverse electric (TE) and magnetic (TM) modes and converts them directly to adsorbed layer mass and thickness by assuming an optical uniform layer and a refractive index varying linearly with mass density [27] upon using Feijter’s Formula [28]. A full description of the technology and measuring principle is included available in [9,10].

### 2.3. Silicon Nitride Deposition and Characterization

Silicon nitride thin films were deposited by r.f. magnetron sputtering (Edwards Coating System ESM100) on titanium oxide gratings and silicon wafer substrates. Film deposition was carried out by sputtering of a pure Si_3_N_4_ target with an r.f. input power of 100 W and using argon as a sputtering gas. The cathode magnetron was 100 mm in diameter, the substrate was rotated in relation with the target and the substrate-target distance was kept at 5 cm. Before deposition, the chamber was evacuated to 1.5 × 10^−5^ mbar and then, backfilled to a working pressure of 5.0 × 10^−3^ mbar. Film thickness and refractive index of the film were determined by ellipsometry (monochrome He-Ne PLASMOS) on the substrates deposited on silicon. The optimal thickness selected for the silicon nitride layer is 10 nm, in order to get a layer thick enough to be uniform and at the same time, thin enough to be transparent and to avoid changing significantly the effective refractive index of the waveguide [11]. For the film required thickness of 10 nm, the sputtering deposition time was established at 3 min.

Silicon nitride coated optical grating coupler sensor chips were characterized by X-Ray Photoemission spectroscopy (XPS) after their activation to be used in immunosensing. XPS spectra were recorded in a Perkin-206 Elmer PHI 5500 Multitechnique System from Physical Electronics (Waltham, MA, USA) with a monochromatic X-ray source (Aluminum KR line of 1,486.6 eV energy and 350 W), placed perpendicular to the analyzer axis and calibrated using the 3d5/2 210 line of Ag with a full width at half-maximum (FWHM) of 0.8 eV. No surface sputtering was used previous to the surface spectra. The resolution selected for the spectra was 187.5 eV of pass energy and 0.8 eV/step. All measurements were taken in an ultra high vacuum (UHV) chamber pressure between 5 × 10^−9^ and 5 × 10^−8^ mbar. When necessary, a low energy electron flood gun (0–3 eV) was used to discharge the samples. Peak fitting was performed using MultiPak 220 V 6.0 A software from Physical Electronics Inc. (Chanhassen, MN, USA). 

### 2.4. Atomic Force Microscopy Surface Characterization

Atomic Force Microscopy (AFM) was used to characterize the topography of the optical gratings before and after the silicon nitride deposition. The measurements were performed in a Dimension 3100 AFM instrument (Veeco Instruments, USA) equipped with a rectangular silicon AFM tip (MikroMasch NSC18/AlBS) with a spring constant of 3.5 N/m, a radius of curvature about 10 nm and a resonance frequency around 75 kHz. The instrument was operated in Tapping mode, in air media and room temperature. The topographic images obtained were analyzed by using the free WSxM software (Nanotec Electrónica, Spain).

### 2.5. Immunosensing Analytical Procedure

**Figure 2:**
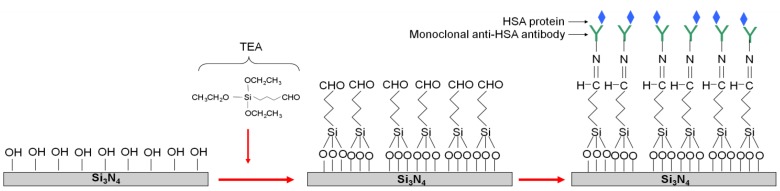
Scheme of the silicon nitride surface functionalization of the direct immobilization of anti-HSA antibodies.

To test the capability of the new structure to work as an immunosensor, standard antibody-antigen detection experiments were performed. The pair anti-HSA/HSA was selected for this purpose, as a model. In order to functionalize the silicon nitride coated OWLS chips for the antibody immobilization, the procedure described by Caballero *et al*. was followed [29] (Figure 2). Briefly, after cleaning with organic solvents and Milli-Q water, the silicon nitride coated chip was immersed in Piranha solution (1:3 v/v H_2_O_2_:H_2_SO_4_; ***Caution:****Piranha is an extremely strong oxidant and should be handled very carefully*) at 90 °C for 30 min. Afterwards, sequential immersion in aqueous solutions of NaOH (0.5 M) for 20 min., HCl (0.1 M) for 10 min. and a final immersion in NaOH (0.5 M) solution for 10 min. were performed to activate the surface. The samples were then rinsed thoroughly with HCl and Milli-Q water and dried in an oven at 100 °C for 20 min. Then, the chip was functionalized with triethoxysilane aldehyde (TEA) by the vapor-phase method for 1 hour and cured into the oven for 1 h at 100 °C. Afterwards, the samples were rinsed with absolute ethanol and dried under nitrogen. Once functionalized with the organosilane, the antibodies were immobilized on the chip surface by immersing it into a 10^−7^ M anti-HSA monoclonal antibody PBS solution (0.01 M phosphate buffer, 2.7 × 10^−3^ M potassium chloride and 0.137 M sodium chloride, pH 8.4), containing 4 mM sodium cyanoborohydride and allowed to react for 1 h at 37 °C. Afterwards, it was thoroughly rinsed with PBS to remove antibody excess. Subsequently, the aldehyde free surface groups were blocked by a solution of ethanolamine (100 mM ethanolamine in 10 mM PBS, pH 8.4) in the presence of 4 mM cyanoborohydride for 1–2 h at room temperature. Finally, the surface was thoroughly rinsed with PBS, pH 8.4. These antibodies will later recognize the HSA protein from the flowing solution.

The OWLS immunosensor system was operated in a continuous flow mode during sample measurement. For the sensing experiments, 500 μL of increasing concentrations (from 10^−13^ M to 10^−3^ M) of the HSA in PBS buffer were injected into the OWLS flow cell. The flow rate of the solution was kept at 30.4 μL/min and the on line sensor response was continuously collected as a surface mass change (ng/cm^2^). After the injection of each analyte concentration, the system was kept in stop-flow mode to favour the analyte incubation for 30 min. Then, the sample was rinsed by flowing PBS solution for 20 min to eliminate unspecific HSA adsorption. The changes of the surface mass during the measurement cycles were automatically calculated by the software implemented in the measurement system through the experimental values of the incoupling angles of the zero transverse electric and magnetic modes. 

## 3. Results and Discussion

### 3.1. Silicon Nitride Thin Layer Characterization

The deposition time and the applied bias of the sputtering process were optimized in order to obtain the best nitride layer in terms of high transmittance in the visible range and in terms of surface smoothness. After deposition, silicon nitride film thickness and refractive index were determined by ellipsometry measurements as 10.3 (±0.1) nm and 2.22 (±0.10), respectively. This refractive index value is higher that the corresponding to a stoichiometric silicon nitride film, which is 2.02 at 300 K [16]. 

**Figure 3:**
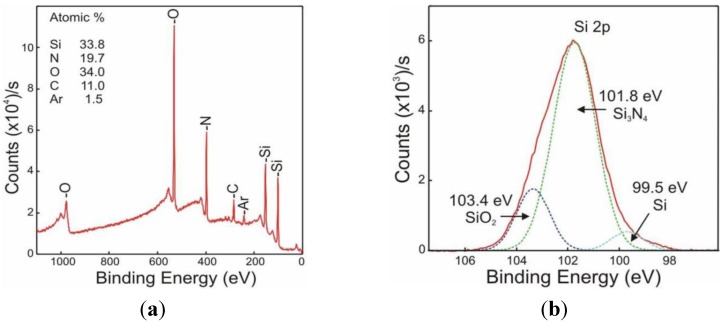
XPS spectra of the activated silicon nitride coated Optical Waveguide Lightmode Spectroscopy (OWLS) chip (**a**) full energy range spectrum and (**b**) high resolution spectrum of the Si 2p peak, showing its deconvolution into three contributions. After the chemical activation procedure with NaOH solution, a high content of O can be observed, which is in accordance with the presence of SiO_2_.

The effects in the chemical composition of the outmost part of the silicon nitride layer produced by the chemical activation process with the NaOH were investigated by means of XPS. Figure 3(a) plots the surface full XPS spectrum of the activated layer, where it can be observed that there is a huge content of oxygen (up to 34% in atomic content). These results agree with the literature, where Si_3_N_4_ surfaces have been oxidized following the same procedure [30]. Moreover, it can be also observed that the ratio Si:N does greatly exceed the stoichiometric ratio of the silicon nitride, being in this case Si_6.8_N_4_. In order to investigate this in more detail, a high-resolution spectrum of the 2p Si peak was performed and analyzed. The peak could be deconvoluted into three contributions (Figure 3(b)), coming from three different oxidation states corresponding to silicon nitride, silicon oxide and silicon [30,31]. These results prove the presence of an oxygen-rich surface that will be then further functionalized by the TEA reagent. 

### 3.2. Silicon Nitride-Coated OWLS Sensor Chip: Atomic Force Microscopy and OWLS Performance Characterization

**Figure 4:**
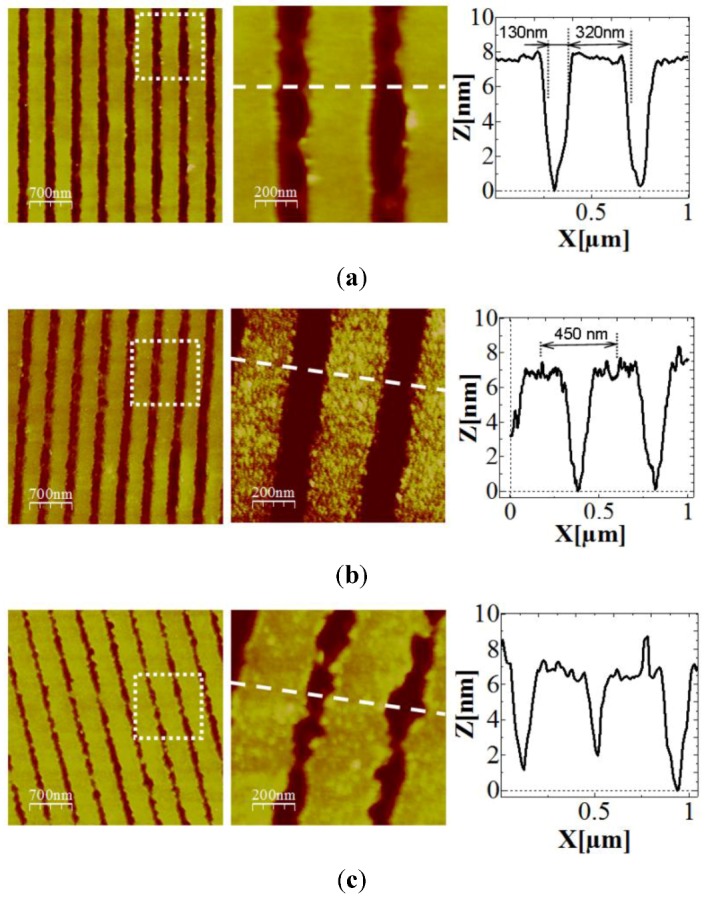
Atomic Force Microscopy (AFM) images of the grating chip (**a**) before, (**b**) after silicon nitride thin layer deposition and (**c**) after antibody functionalization. The grating structure is maintained after both the silicon nitride thin film and the organic layer deposition.

Prior to the silicon nitride layer deposition, the grating substrate was characterized by means of atomic force microscopy (AFM). Figure 4(a) shows 2D topography AFM images of the grating and a line profile. As it can be seen, the grating has a period of 450 nm, a grafting apparent depth of around 7 nm and a RMS value on the flat top surfaces of 0.7 nm. Due to the AFM tip convolution, it is likely that the measured depth of the grating is underestimated. After the deposition of the silicon nitride layer, Figure 4(b), it can be noticed that surface roughness has increased up to 2.6 nm (RMS value), presenting a grainier morphology, while the grating structure remained barely altered (period of 450 nm and apparent depth of 7 nm). Once the chip was functionalized with the anti-HSA antibodies, the morphology of the grating was again characterized by the AFM technique. Figure 4(c) shows the changes produced by the organic layer: the grating structure period remains unaltered (450 nm) but the structure depth decreases slightly (5–6 nm) and the surface becomes again smoother, with RMS values around 1.8 nm.

**Figure 5:**
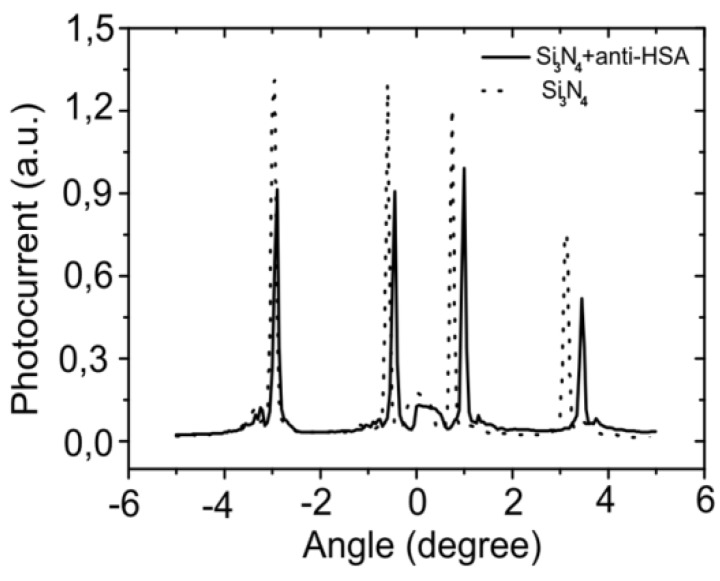
The laser beam is coupled inside the waveguide at different incoupling angles (depending on the covering layer). The spectrum is followed by the photodiodes placed at each end of the waveguide. The dash line shows the incoupling spectra of the bare chip covered with silicon nitride, and the continuous line after antibody functionalization.

Once fabricated, the functionalized silicon nitride chip performance in the OWLS instrument was checked. Figure 5 shows the plot of OWLS incoupling angle spectra after the silicon nitride layer deposition. It can be seen that both TE and TM peaks are perfectly visible after the silicon nitride deposition and the signal to noise ratio increases from 41 to 56 in the OWLS spectra when the chip is functionalized with antibodies with respect to the bare Si_3_N_4_ coated chip. This is related to the decrease in the roughness as a flatter surface avoids scattering during the incoupling of the laser. The same figure shows the OWLS incoupling angle peaks after the chip functionalization with the anti-HSA antibody layer. Worth noticing, the functionalization of the chip did not affect the chip structure neither covered the whole chip surface, allowing the coupling of the laser. The chips may be used a minimum of three times, maintaining their properties before the nitride layer gets damaged.

### 3.3. Immunosensor: Proof of Concept

**Figure 6:**
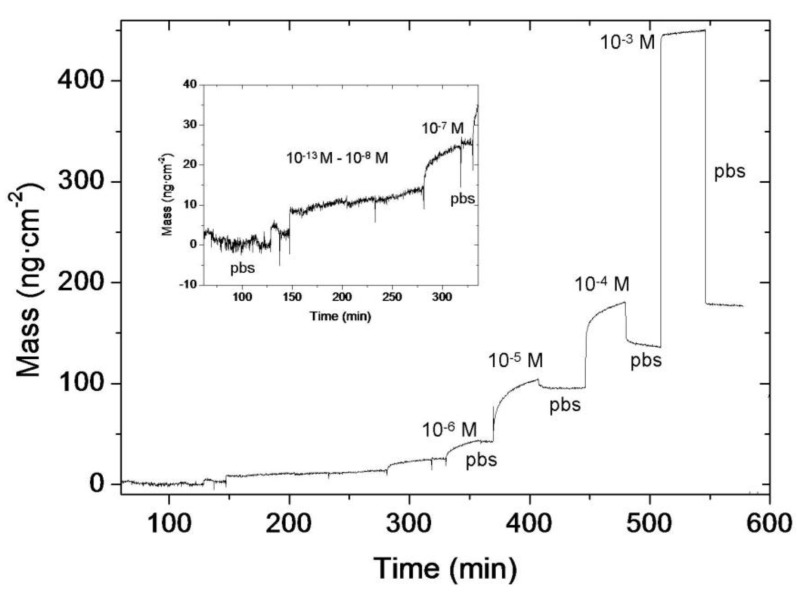
OWLS graphic showing the mass of HSA protein adsorbed on the Si_3_N_4_ functionalized chip.

Human serum albumin detection experiments were performed in order to establish the biosensing viability of the whole developed system. The albumin concentration in the blood serum of an average adult human is 42.0 ± 3.5 mg/mL, (~0.6 mM) with an interval of 35–50 mg/mL [32,33]. Hyperalbuminemia and hypoalbuminemia are pathogenesis related with high or low HSA concentrations in body fluids, respectively. The immunosensing experiments have been specifically design to show high sensitivity and dynamic range performance in the range of interest. HSA was recognized by the anti-HSA antibody attached to the silicon nitride coated OWLS waveguide chip. The system proved to be stable; responses did not decrease significantly during the measurement. A typical curve of the measurements performed with the system is shown in Figure 6. Measurements started by stabilizing the system by flowing PBS for two hours approximately in order to obtain a steady baseline. Then, solutions with increasing HSA protein concentrations within the range of 10^−13^ M–10^−3^ M were injected in the cell. After each injection, the system was kept in stop-flow mode in order to stabilize the signal, and then it was thoroughly rinsed with PBS in order to remove all the protein non-specifically adhered to the chip surface. The system response was sigmoidal with the protein concentration, as predicted by the Nerst equation and the Langmuir adsorption theory [34]. The system proved to be very stable, achieving repeatable results with an error of less than 10%. Considering the amount of antibody captured probes used on the immobilization procedure (equivalent to 104 pmol/cm^2^) and taking into account that an antibody perfect monolayer would have a density of is 1.7 pmol/cm^2^ (antibodies are around 10 nm in diameter [35]) the antibody amount used in the immobilization process is well in excess. If the recognition ratio between the protein and the antibody is 0.37:1 [36], the maximum saturation value of protein to be recognised for an ideal antibody monolayer would be 336 ng/cm^2^. Thus, the experimental data measured for the adsorbed protein in the detection range assayed, shown in Figure 6, is far from saturation, therefore confirming that the biosensing system is well designed to work in the desired range.

**Figure 7:**
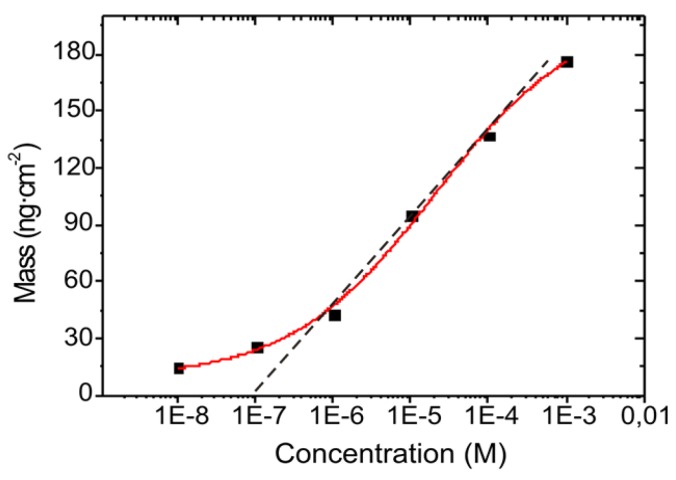
Relationship between the adsorbed mass of HSA protein in the surface and the HSA solution concentration. The dash line shows a linear fit for the dynamic range of the sensor.

The relationship between the adsorbed mass, attributed to the HSA protein and the protein concentration plotted in a semi-logarithmic scale is shown in Figure 7. The experimental mass values were fitted using a sigmoidal fit with a correlation factor of R^2^ = 0.9969. The graphic also suggests a linear fit within the range of 10^−3^ M to 10^−6^ M with a R^2^ value of 0.99689. The sensitivity of the system within the dynamic range is 34 ng/cm^2^ for the HSA detection. 

These results show a biosensing system with a dynamic working range particularly well fitted and designed for the specific application, with a LOD that, despite not reaching the values reported by similar studies [37], is three orders of magnitude better than needed for diagnosis of hyper/hypoalbuminemia and far more stable. Furthermore, our system presents a very good sensitivity and the possibility of extracting values of adsorbed mass, in contrast with previous studies [37] where was only possible to measure changes in refractive index units (RIU). This model study of the detection and quantification of HSA, demonstrate the potential of the Si_3_N_4_ covered grating sensor chips. The ability of the silicon nitride to be the functional layer in a real-time biosensor, renders it a promising material for integration of optical waveguides with microelectronics; for instance, the integration of optical waveguides into printed-circuit boards, or into field effect transistors.

## 4. Conclusions

An Optical Waveguide Lightmode Spectroscopy (OWLS) chip with a silicon nitride grating surface has been fabricated by coating a diffraction grating with a 10 nm thin layer of Si_3_N_4_ by sputtering deposition, confirmed by AFM, ellipsometry and XPS measurements. A novel protocol was used for the direct immobilization of biomolecules onto the silicon nitride optical immunosensor and a self-assembled layer of anti-human serum albumin antibodies were directly immobilized on the functionalized Si_3_N_4_ OWLS chip for the detection of different concentrations of HSA proteins, as a proof of concept. Results show that the adsorbed mass of protein can be easily measured, giving extra information about the molecular interactions processes compared with previous detection techniques; for instance the amount of protein immobilized on the surface. The potential of the Si_3_N_4_ covered grating sensor chips opens new ways in biosensing, allowing the integration of optical biosensors with microelectronics.
